# Global hydro-environmental sub-basin and river reach characteristics at high spatial resolution

**DOI:** 10.1038/s41597-019-0300-6

**Published:** 2019-12-09

**Authors:** Simon Linke, Bernhard Lehner, Camille Ouellet Dallaire, Joseph Ariwi, Günther Grill, Mira Anand, Penny Beames, Vicente Burchard-Levine, Sally Maxwell, Hana Moidu, Florence Tan, Michele Thieme

**Affiliations:** 1 Australian Rivers Institute, Griffith University, Brisbane, QLD 4111 Australia; 2 Department of Geography, McGill University, Montreal, QC H3A 0B9 Canada; 3 World Wildlife Fund, Washington, DC 20037 USA

**Keywords:** Freshwater ecology, Hydrology

## Abstract

The HydroATLAS database provides a standardized compendium of descriptive hydro-environmental information for all watersheds and rivers of the world at high spatial resolution. Version 1.0 of HydroATLAS offers data for 56 variables, partitioned into 281 individual attributes and organized in six categories: hydrology; physiography; climate; land cover & use; soils & geology; and anthropogenic influences. HydroATLAS derives the hydro-environmental characteristics by aggregating and reformatting original data from well-established global digital maps, and by accumulating them along the drainage network from headwaters to ocean outlets. The attributes are linked to hierarchically nested sub-basins at multiple scales, as well as to individual river reaches, both extracted from the global HydroSHEDS database at 15 arc-second (~500 m) resolution. The sub-basin and river reach information is offered in two companion datasets: BasinATLAS and RiverATLAS. The standardized format of HydroATLAS ensures easy applicability while the inherent topological information supports basic network functionality such as identifying up- and downstream connections. HydroATLAS is fully compatible with other products of the overarching HydroSHEDS project enabling versatile hydro-ecological assessments for a broad user community.

## Abstract (summary)

**Table:** 

Measurement(s)	hydro-environmental characteristics • watershed • drainage basin • river • stream • hydrographic feature
Technology Type(s)	digital curation
Sample Characteristic - Environment	freshwater environment • aquatic environment
Sample Characteristic - Location	Earth (planet)

Machine-accessible metadata file describing the reported data: 10.6084/m9.figshare.10059551

## Background & Summary

Freshwater systems are under multiple threats^1^ which can be detrimental to their biodiversity and the ecosystem services they provide^2–5^. Researchers, governments, water managers, policy makers, and conservation organizations around the world face the challenge of developing innovative strategies to alleviate the pressures on freshwater resources^6^, and many applied approaches and solutions require large amounts of data^7^. Furthermore, integrated freshwater resource assessments are often carried out at large scales, from regional to global, and thus suffer from incompatible or differing data conventions among the involved spatial units, such as multiple countries or river basins. More than 260 large basins are considered transboundary at the global scale, representing 45% of the land surface and 40% of the world’s population^8^. In these cases, global data can provide consistent and homogeneous coverage required for seamless analyses. Global data can also provide baseline information in remote areas where little monitoring is available yet stakeholders need to address urgent issues in a timely manner.

Significant progress has been made in recent years in the creation of increasingly high-resolution and accurate hydrographic information that allows the delineation of watershed boundaries and river networks from global digital elevation models (DEMs) at up to ~90 m pixel resolution, with arguably the most prominent example being the HydroSHEDS database (Hydrological data and maps based on SHuttle Elevation Derivatives at multiple Scales)^9^. Despite these advancements, users interested in additional watershed or river characteristics, such as topographic, climatic or land cover information, are required to derive or summarize these data independently from alternative sources. This typically involves repetitive geospatial procedures that assign the attribute values of auxiliary datasets to the desired sub-basin or river units, often necessitating the development of new algorithms or software customizations within Geographic Information Systems (GIS). Besides the time-consuming processing, the individual, non-standardized solutions create results that are difficult to compare.

To offer consistent baseline data without the need of repetition, efforts have been made in the past to create predefined compilations of hydro-environmental watershed and river characteristics. Prominent national examples include the Australian Hydrological Geospatial Fabric (Geofabric; http://www.bom.gov.au/water/geofabric)^10^ which is built upon a stream and nested catchment framework at a spatial resolution of 9 arc-seconds (~270 m)^11^ and is accompanied by nearly 400 attributes describing the natural and anthropogenic environment of approximately 1.4 million river reaches and sub-catchments at multiple scales. In the Unites States, the National Hydrography Database (NHD; https://nhd.usgs.gov)^12^ provides a geospatial surface water framework and has become a highly-valued information resource for water-related applications. It incorporates different baseline datasets at varying scales and resolutions both in vector and raster format, and the value added attributes (VAAs) of the enhanced NHDPlus (http://www.horizon-systems.com/NHDPlus/NHDPlusV2_home.php)^13^ expand the capabilities for upstream and downstream navigation, analysis, and modeling. This was further augmented by the StreamCat dataset (https://www.epa.gov/national-aquatic-resource-surveys/streamcat)^14^ which offers more than 100 variables for predicting aquatic conditions and watershed integrity. Similarly, the European Catchments and Rivers Network System (ECRINS)^15^, based on the Catchment Characterisation and Modelling project (CCM2)^16^, provides a dynamic set of map layers and river catchment information designed to support environmental analyses and policy-making, including the implementation of the EU Water Framework Directive.

At even larger scales, Domisch *et al*.^17^ presented a near-global, spatially continuous, and freshwater-specific set of environmental variables for a standardized 1 km river network grid. They derived more than 300 individual attributes of climatic, stream-topographic, land cover, geological, and soil characteristics and applied upstream accumulation techniques to assess the watershed contributions to each river pixel. Although their river network is based on the HydroSHEDS database^9^, they applied local modifications which render the results unique to their own flow directional grid. Also, they do not provide a sub-basin perspective, and the spatial extent is limited to below 60 degrees northern latitude.

To enhance global spatial coverage and standardization, we here introduce the HydroATLAS database. HydroATLAS provides a single, comprehensive, consistently organized and fully-global data compendium that gathers and presents a wide range of hydro-environmentally relevant characteristics at both sub-basin and river reach scales at high spatial resolution. The hydro-environmental attributes are compiled from publicly available data sources and are organized in six categories: hydrology; physiography; climate; land cover & use; soils & geology; and anthropogenic influences (Table 1).

**Table 1:** Categories of hydro-environmental characteristics included in the HydroATLAS database.

Category	Description
Hydrology & hydrography	Hydrological and hydrographic characteristics related to quantity, quality, location and extent of terrestrial water *Examples: natural annual runoff and discharge*, *lake cover*, *groundwater table depth*
Physiography	Topographic and geomorphic characteristics related to terrain, relief or landscape position *Examples: elevation*, *slope*, *and derivatives*
Climate	Climatic characteristics *Examples: mean temperature/precipitation/evaporation*, *climate moisture index*, *global aridity*
Land cover & use	Land cover and land use characteristics including biogeographic regions *Examples: land cover classes*, *permafrost extent*, *terrestrialand* *freshwater ecoregions*
Soils & geology	Soil and geology related characteristics including substrate types and soil conditions *Examples: percent sand/silt/clay in soil*, *soil water stress*, *lithography*, *karst*, *soil erosion*
Anthropogenic influences	Anthropogenic characteristics and influences including demographic and socioeconomic aspects *Examples: population density*, *human footprint*, *GDP per capita*

**Fig. 1:**
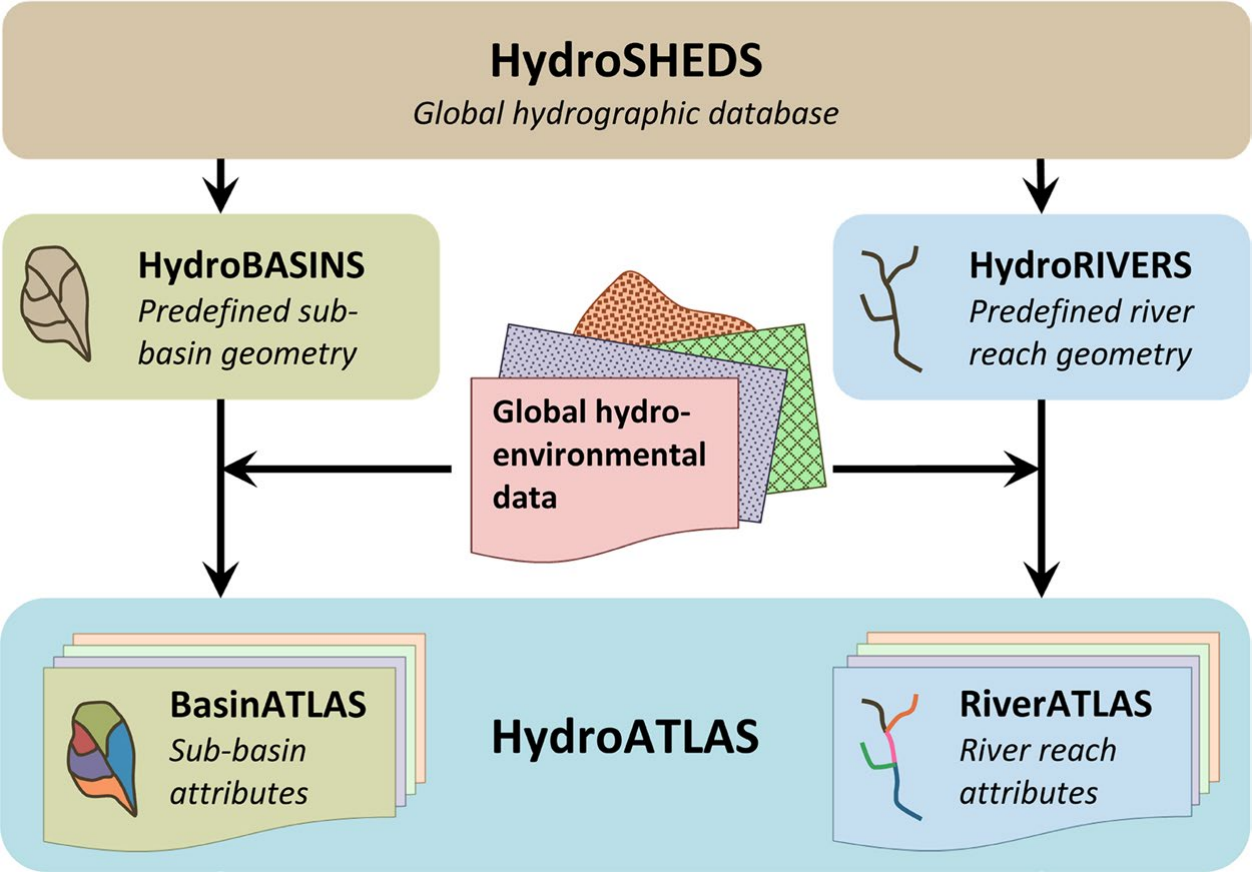
Conceptual design of HydroATLAS and relationship to underpinning HydroSHEDS database.

HydroATLAS consists of two companion attribute databases that have been created in tandem (Fig. 1). The first database, BasinATLAS, derives sub-basin characteristics for hierarchically nested watersheds at twelve spatial scales. The second database, RiverATLAS, provides similar attributes yet derived for river and stream reaches rather than sub-basins. The geospatial units for both databases, i.e. sub-basin polygons and river reach line segments, respectively, have been derived from the global hydrographic database HydroSHEDS^9^ at a spatial resolution of 15 arc-seconds (~500 m at the equator). For this purpose, two predefined geometry datasets were extracted from HydroSHEDS: a sub-basin geometry dataset in polygon format termed HydroBASINS, consisting of twelve individual layers representing nested sub-basin scales; and a river reach geometry dataset in line format termed HydroRIVERS, consisting of a single layer.

**Fig. 2:**
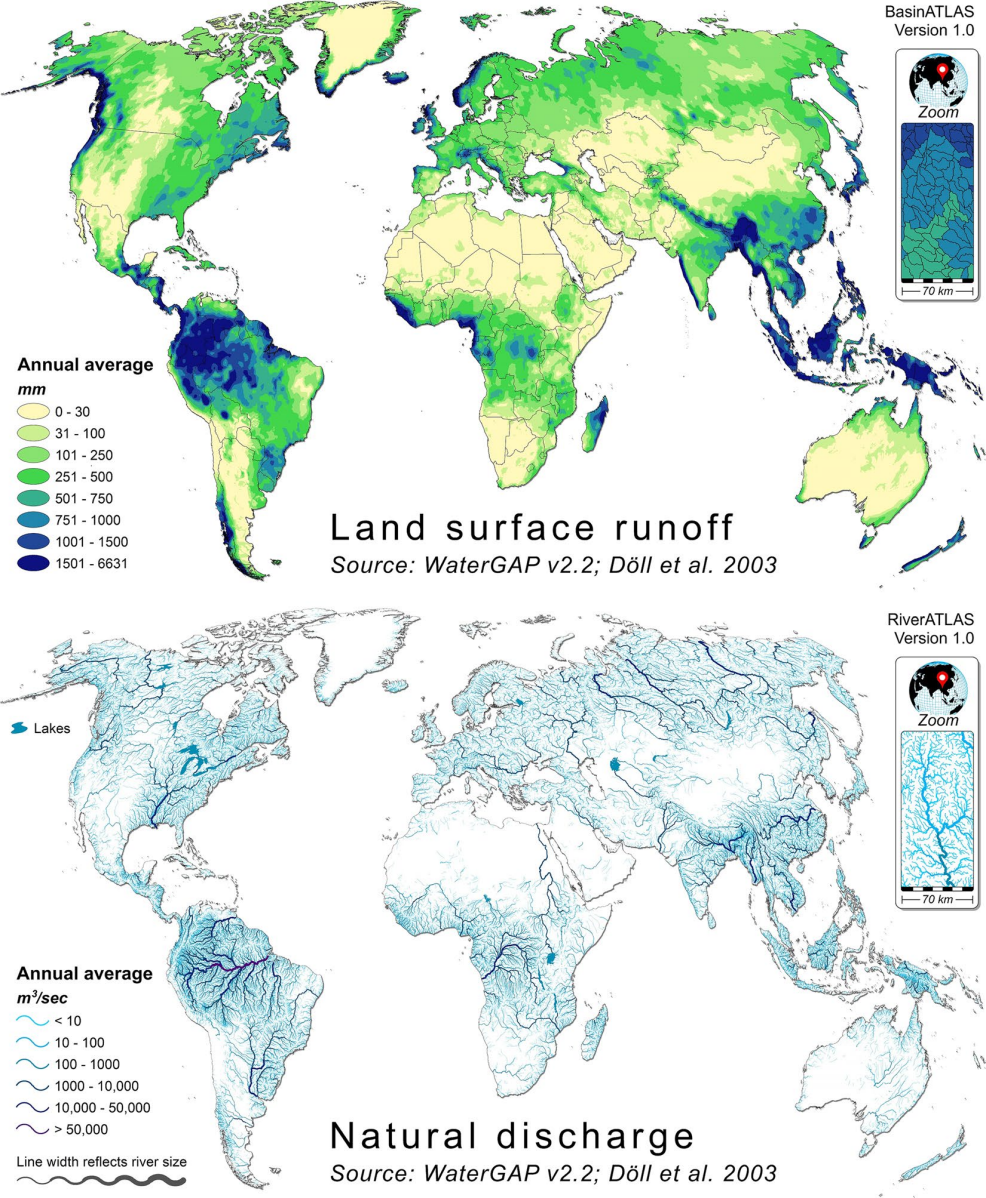
Example attributes of HydroATLAS. Top panel: land surface runoff per sub-basin of BasinATLAS (level 10 subdivisions). Bottom panel: natural discharge per river reach of RiverATLAS.

Version 1.0 of HydroATLAS offers a total of 281 individual attributes, representing 56 different hydro-environmental variables (Table 2), each associated with the twelve sub-basin polygon layers of BasinATLAS and the line segments of RiverATLAS (Fig. 2). At its highest level of subdivision, BasinATLAS contains 1.0 million sub-basins with an average area of 130.6 km^2^, representing a total of 135.0 million km^2^ of global land area (excluding Antarctica). RiverATLAS encompasses 8.5 million line segments with an average length of 4.2 km, representing a total of 35.9 million km of rivers globally. HydroATLAS is envisioned to be expanded and updated in the future with new attribute data as more global information becomes available, or by customizing it for individual regional applications.

**Table 2:** Hydro-environmental attributes provided in version 1.0 of the HydroATLAS database.

ID	Category	Variable	Source data	Source Resolution (G: Grid V: Vector)	Source year	Reference	Number/type of individual attributes		
							General*	Monthly	Upstream
H01	Hydrology	Natural Discharge	WaterGAP v2.2	G: 15 arc-sec	1971–2000^+^	Döll *et al*.^33^	3		
H02	Hydrology	Land Surface Runoff	WaterGAP v2.2	G: 15 arc-sec	1971–2000^+^	Döll *et al*.^33^	1		
H03	Hydrology	Inundation Extent	GIEMS-D15	G: 15 arc-sec	1993–2004	Fluet-Chouinard *et al*.^39^	3		3
H04	Hydrology	Limnicity (percent lake area)	HydroLAKES	V: ~1: 250,000	most recent^$^	Messager *et al*.^37^	1		1
H05	Hydrology	Lake Volume	HydroLAKES	V: ~1: 250,000	most recent^$^	Messager *et al*.^37^			1
H06	Hydrology	Reservoir Volume	GRanD v1.1	V: ~1: 1 million	most recent^$^	Lehner *et al*.^40^			1
H07	Hydrology	Degree of Regulation	HydroSHEDS & GRanD	G: 15 arc-sec	most recent^$^	Lehner *et al*.^40^	1		
H08	Hydrology	River Area	HydroSHEDS & WaterGAP	G: 15 arc-sec	1971–2000^+^	Lehner & Grill^26^	1		1
H09	Hydrology	River Volume	HydroSHEDS & WaterGAP	G: 15 arc-sec	1971–2000^+^	Lehner & Grill^26^	1		1
H10	Hydrology	Groundwater Table Depth	Global Groundwater Map	G: 30 arc-sec	1927–2009^+^	Fan *et al*.^41^	1		
P01	Physiography	Elevation	EarthEnv-DEM90	G: 3 arc-sec	2000–2010	Robinson *et al*.^42^	3		1
P02	Physiography	Terrain Slope	EarthEnv-DEM90	G: 3 arc-sec	2000–2010	Robinson *et al*.^42^	1		1
P03	Physiography	Stream Gradient	EarthEnv-DEM90	G: 3 arc-sec	2000–2010	Robinson *et al*.^42^	1		
C01	Climate	Climate Zones	GEnS	G: 30 arc-sec	2000	Metzger *et al*.^43^	1		
C02	Climate	Climate Strata	GEnS	G: 30 arc-sec	2000	Metzger *et al*.^43^	1		
C03	Climate	Air Temperature	WorldClim v1.4	G: 30 arc-sec	1950–2000	Hijmans *et al*.^44^	3	12	1
C04	Climate	Precipitation	WorldClim v1.4	G: 30 arc-sec	1950–2000	Hijmans *et al*.^44^	1	12	1
C05	Climate	Potential Evapotranspiration	Global-PET	G: 30 arc-sec	1950–2000^+^	Zomer *et al*.^45^; Trabucco *et al*.^46^	1	12	1
C06	Climate	Actual Evapotranspiration	Global Soil-Water Balance	G: 30 arc-sec	1950–2000^+^	Trabucco & Zomer^47^	1	12	1
C07	Climate	Global Aridity Index	Global Aridity Index	G: 30 arc-sec	1950–2000^+^	Zomer *et al*.^45^; Trabucco *et al*.^46^	1		1
C08	Climate	Climate Moisture Index	WorldClim & Global-PET	G: 30 arc-sec	1950–2000^+^	Hijmans *et al*.^44^; Zomer *et al*.^45^	1	12	1
C09	Climate	Snow Cover Extent	MODIS/Aqua	G: 15 arc-sec	2002–2015	Hall & Riggs^48^	2	12	1
L01	Land cover/use	Land Cover Classes	GLC2000	G: 30 arc-sec	2000	Bartholomé & Belward^49^	1		
L02	Land cover/use	Land Cover Extent	GLC2000	G: 30 arc-sec	2000	Bartholomé & Belward^49^	22		22
L03	Land cover/use	Potential Natural Vegetation Classes	EarthStat	G: 5 arc-min	1700	Ramankutty & Foley^50^	1		
L04	Land cover/use	Potential Natural Vegetation Extent	EarthStat	G: 5 arc-min	1700	Ramankutty & Foley^50^	15		15
L05	Land cover/use	Wetland Classes	GLWD	G: 30 arc-sec	historic	Lehner & Döll^51^	1		
L06	Land cover/use	Wetland Extent	GLWD	G: 30 arc-sec	historic	Lehner & Döll^51^	11		11
L07	Land cover/use	Forest Extent	GLC2000	G: 30 arc-sec	2000	Bartholomé & Belward^49^	1		1
L08	Land cover/use	Cropland Extent	EarthStat	G: 5 arc-min	2000	Ramankutty *et al*.^52^	1		1
L09	Land cover/use	Pasture Extent	EarthStat	G: 5 arc-min	2000	Ramankutty *et al*.^52^	1		1
L10	Land cover/use	Irrigated Area Extent	HID v1.0	G: 5 arc-min	2005	Siebert *et al*.^53^	1		1
L11	Land cover/use	Glacier Extent	GLIMS	V: unspecified	1950–2015	GLIMS & NSIDC^54^	1		1
L12	Land cover/use	Permafrost Extent	PZI	G: 30 arc-sec	1961–1990^+^	Gruber^55^	1		1
L13	Land cover/use	Protected Area Extent	WDPA	V: varying	most recent^$^	UNEP-WCMC & IUCN^56^	1		1
L14	Land cover/use	Terrestrial Biomes	TEOW	V: ~1: 1 million	most recent^$^	Dinerstein *et al*.^57^	1		
L15	Land cover/use	Terrestrial Ecoregions	TEOW	V: ~1: 1 million	most recent^$^	Dinerstein *et al*.^57^	1		
L16	Land cover/use	Freshwater Major Habitat Types	FEOW	V: ~1: 1 million	most recent^$^	Abell *et al*.^58^	1		
L17	Land cover/use	Freshwater Ecoregions	FEOW	V: ~1: 1 million	most recent^$^	Abell *et al*.^58^	1		
S01	Soils &Geology	Clay Fraction in Soil	SoilGrids1km	G: 30 arc-sec	most recent^+^	Hengl *et al*.^59^	1		1
S02	Soils & Geology	Silt Fraction in Soil	SoilGrids1km	G: 30 arc-sec	most recent^+^	Hengl *et al*.^59^	1		1
S03	Soils & Geology	Sand Fraction in Soil	SoilGrids1km	G: 30 arc-sec	most recent^+^	Hengl *et al*.^59^	1		1
S04	Soils & Geology	Organic Carbon Content in Soil	SoilGrids1km	G: 30 arc-sec	most recent^+^	Hengl *et al*.^59^	1		1
S05	Soils & Geology	Soil Water Content	Global Soil-Water Balance	G: 30 arc-sec	1950–2000^+^	Trabucco & Zomer^47^	1	12	1
S06	Soils & Geology	Lithological Classes	GLiM	G: 0.5 degrees	1965–2012	Hartmann & Moosdorf^60^	1		
S07	Soils & Geology	Karst Area Extent	Rock Outcrops v3.0	V: unspecified	most recent^$^	Williams & Ford^61^	1		1
S08	Soils & Geology	Soil Erosion	GloSEM v1.2	G: 7.5 arc-sec	2012	Borrelli *et al*.^62^	1		1
A01	Anthropogenic	Population Count	GPW v4	G: 30 arc-sec	2010	CIESIN & SEDAC^34^	1		1
A02	Anthropogenic	Population Density	GPW v4	G: 30 arc-sec	2010	CIESIN & SEDAC^34^	1		1
A03	Anthropogenic	Urban Extent	GHS S-MOD v1.0 (2016)	G: 1 km	2015^+^	Pesaresi & Freire^63^	1		1
A04	Anthropogenic	Nighttime Lights	Nighttime Lights v4	G: 30 arc-sec	2008	Doll^64^	1		1
A05	Anthropogenic	Road Density	GRIP v4	G: 5 arc-min	> 1997^$^	Meijer *et al*.^65^	1		1
A06	Anthropogenic	Human Footprint	Human Footprint v2	G: 1 km	1993 & 2009	Venter *et al*.^66^	2		2
A07	Anthropogenic	Global Administrative Areas	GADM	V: unspecified	2012	University of Berkeley^67^	1		
A08	Anthropogenic	Gross Domestic Product	GDP PPP v2	G: 5 arc-min	2015	Kummu *et al*.^68^	2		1
A09	Anthropogenic	Human Development Index	HDI v2	G: 5 arc-min	2015	Kummu *et al*.^68^	1		
		**Σ** = **56 (Variables)**				**Σ** = **281 (Attributes)**	**110**	**84**	**87**

*May include different attributes, for example individual classes, average, minimum, and/or maximum values.

^$^Data have been compiled from various sources with varying or unknown dates, but are supposed to resemble contemporary/most recent conditions.

^+^Model-based.

The HydroATLAS database is expected to create novel opportunities of multi-variable statistical assessments or model-based analyses for a mix of theoretical and applied hydro-ecological studies, and to offer a particular research stimulus in otherwise data poor and/or remote regions. For example, HydroATLAS can facilitate large-scale assessments of the environmental conditions of watersheds or river networks and has already been used to support systematic classification efforts at sub-basin and river reach scales^18,19^ and to measure the ‘free-flowing’ status of all rivers globally^20^. Other key applications in ecological sciences include species distribution modelling and conservation planning^21–24^. We also imagine advances in macro-ecology, such as exploring life history traits or environmental drivers, as other global databases containing functional ecological parameters become available that can be combined with HydroATLAS. Examples for such linkages to auxiliary ecological information could include regional or global fish distributions from the International Union for Conservation of Nature (IUCN)^25^; data sourced from FISHBASE (http://www.fishbase.org) or the COMPADRE/COMADRE plant and animal matrix databases (http://www.compadre-db.org). The corresponding spatial relationships, once established, are expected to further amplify the utility and versatility of the HydroATLAS database.

## Methods

### Global underpinning hydrography of HydroSHEDS

The spatial sub-basin and river reach geometry used in HydroATLAS is derived from the global HydroSHEDS database^9^. HydroSHEDS provides hydrographic baseline information in a consistent and comprehensive format to support regional and global watershed analyses, hydrological modeling, and freshwater conservation planning. It is currently considered the leading global product in terms of quality and resolution^11,26^. HydroSHEDS offers a suite of geo-referenced datasets at multiple scales as seamless global coverages, including both raster and vector formats. The core data layers are a hydrologically conditioned digital elevation model and a corresponding drainage direction map from which auxiliary layers can be derived, including flow accumulations, flow distances, river orders, watershed boundaries, and stream networks. HydroSHEDS was initially derived from elevation data of the Shuttle Radar Topography Mission (SRTM)^27,28^ at a pixel resolution of 3 arc-seconds (~90 m at the equator) and was subsequently upscaled to resolutions of 15 and 30 arc-seconds (~500 m and 1 km at the equator, respectively). More information on HydroSHEDS is provided at http://www.hydrosheds.org.

### Global sub-basin geometry of HydroBASINS

**Fig. 3:**
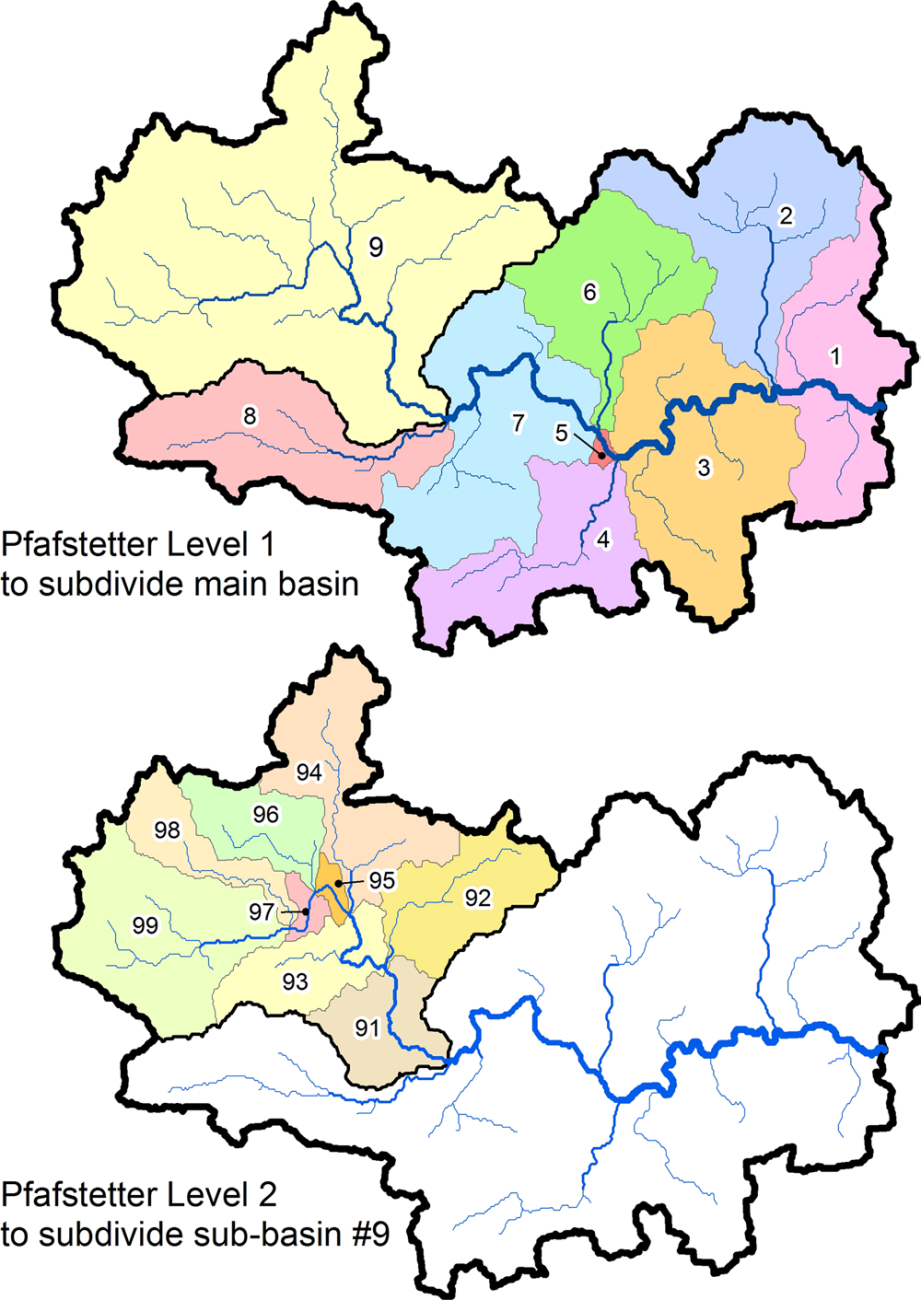
Overview of the Pfafstetter coding scheme used in the HydroBASINS dataset. At the first level (top panel), the original watershed is divided into nine sub-basins (i.e., into the four largest tributaries and the five resultant inter-basins). At the next level (bottom panel), each sub-basin is again divided into nine sub-basins. This process is iterated for each subsequent level of subdivisions.

Basins and sub-basins have been preprocessed as a customized derivative of HydroSHEDS and are offered as a stand-alone product termed HydroBASINS^26,29^. Based on the HydroSHEDS drainage direction map at 15 arc-second resolution, watershed boundaries were delineated and subdivided following the topological concept of the Pfafstetter coding system^30^ which provides a methodology for the breakdown of sub-basins into increasingly smaller sizes in a hierarchical and systematic manner (Fig. 3). Following this coding scheme, twelve nested levels of sub-basins were generated globally, each depicting consistently sized sub-basin polygons at scales ranging from millions (level 1) to tens of square kilometers (level 12).

Connectivity between sub-basins is defined based on the underpinning drainage direction map of HydroSHEDS which identifies the ID of the next downstream neighbor of every sub-basin (except for those sub-basins ending at the ocean or at inland sinks). The HydroBASINS dataset does not contain any hydro-environmental attribute information other than what can be derived directly from the polygon geometry and topology, including the polygon area and the total upstream contributing watershed area.

### Global river reach geometry of HydroRIVERS

**Fig. 4:**
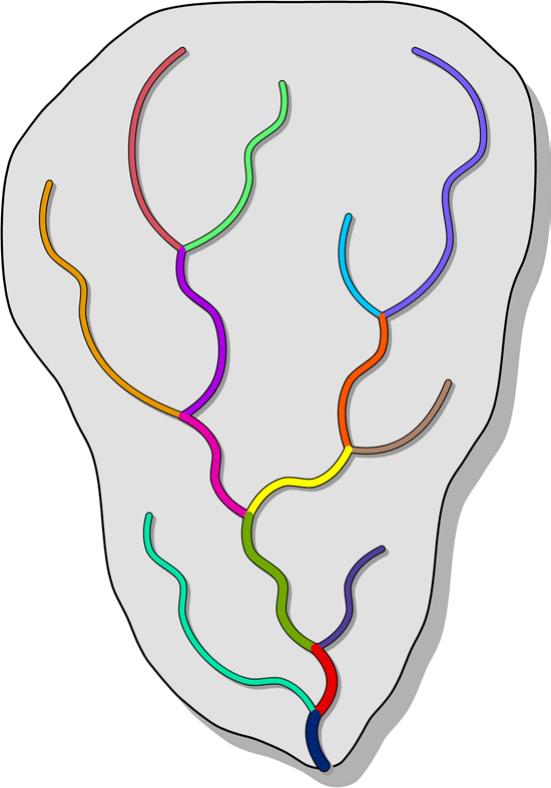
Overview of the river reach concept used in the HydroRIVERS dataset. Every river reach, depicted by a line segment in a different color, is defined as a stretch of river between two tributaries, or between the start/end of the network and a tributary.

A global river network delineation has been extracted from HydroSHEDS at 15 arc-second resolution and is available as a stand-alone vector product termed HydroRIVERS (see http://www.hydrosheds.org). For this network, rivers have been defined to start at all pixels where the accumulated upstream watershed area exceeds 10 km^2^, or where the long-term average natural discharge exceeds 0.1 cubic meters per second (for more details on the quality of the discharge data see *Technical Validation* below), or both. Streams smaller than these thresholds were not extracted as they are increasingly unreliable in their spatial representation due to the uncertainties in the underpinning global geometric and hydrologic data. All identified river pixels at 15 arc-second resolution were then converted into vector format to produce a line network consisting of individual river reaches (Fig. 4). It should be noted that we here define a ‘river reach’ as a simple cartographic unit, i.e. the line segment between two neighbouring confluences, rather than a functional unit that encompasses certain ecosystem processes or habitats.

Connectivity between reaches is defined based on the underpinning drainage direction map of HydroSHEDS which identifies the ID of the next downstream neighbor to every reach (except for those reaches ending at the ocean or at inland sinks). The HydroRIVERS dataset does not contain any hydro-environmental attribute information other than what can be derived directly from the line geometry and topology, including the length of the river reach; the distance from the upstream headwater source and from the final downstream pour point; and the upstream contributing watershed area.

### Acquisition and selection of hydro-environmentally relevant attribute data

Raster or vector input data for all hydro-environmental characteristics were acquired either from free and publicly available sources, or from collaborators who provided their data for this project. All data sources were assessed regarding their suitability for this project using the following selection criteria:

- completeness of global coverage (allowing only for minor spatial gaps, such as small remote islands, or omission of non-critical areas, such as Greenland or deserts);
- consistency in data quality (i.e., no regional or local biases);
- sufficiency of the native resolution, precision and accuracy (e.g., if the original pixel size is exceeding the size of sub-basins at the smallest level of subdivision, it is generally deemed inappropriate for the derivation of sub-basin attributes); and
- permission to use and distribute derivatives under a free license.

If multiple datasets were available for the same attribute, priority was given to the most widely recognized and/or best resolution and/or most recent dataset. It should be noted, however, that the selection of an attribute dataset does not imply any kind of endorsement or warranty of its quality or superiority over other data.

### Preprocessing of attribute data

Before extracting their attribute information into HydroATLAS format, the original attribute datasets were preprocessed into a standardized grid format with the same geometric specifications as the HydroSHEDS 15 arc-second resolution grids. The goal of this step was to ensure full spatial congruency between (preprocessed) attribute data and HydroSHEDS to avoid misalignments in the subsequent conversion processes. Accordingly, the target specifications were: a global extent of 180°W to 180°E in longitude and 84°N to 56°S in latitude; a cell size of 15 arc-seconds; a global projection defined by the Geographic Coordinate System with the horizontal datum of the World Geodetic System 1984 (GCS_WGS_1984); and a land-ocean distribution of pixels following the land mask of HydroSHEDS. General preprocessing methods are described below; additional details, including the format and resolution of each individual attribute dataset, are provided in the Technical Documentation accompanying the HydroATLAS data (http://www.hydrosheds.org/page/hydroatlas).

If required, original data were first re-projected into the GCS_WGS_1984 coordinate system. If an original dataset was in grid format with a cell size other than 15 arc-seconds, it was either aggregated or disaggregated, depending on its native resolution. For disaggregation, original attribute values were preserved, i.e. each large cell was simply subdivided into smaller pixels using ‘nearest neighbor’ sampling, unless the data type necessitated a value conversion (e.g., to preserve original population numbers, the total population count of a large cell was divided by the number of resulting sub-pixels it was split into). For aggregation, an appropriate summary statistic was calculated; this was typically the ‘average’ for continuous data such as elevation, and the ‘majority’ for categorical data such as land cover types. For certain high resolution categorical datasets, a new attribute was calculated representing the percent coverage of a class within each 15 arc-second pixel (e.g., percent lake cover). If an interpolation was required during the re-projection, disaggregation or aggregation process of grids, which was only the case if the original resolution was a non-integer factor or divisor of the target 15 arc-second resolution, ‘nearest neighbor’ interpolation was applied to avoid alteration of original values.

If original datasets were in vector format, i.e. representing data as points, lines, or polygons, these were converted to grids of the same extent and pixel size as the HydroSHEDS data. If the original vector maps offered sufficient precision, the data were first converted to a grid of higher resolution, e.g. tenfold at 1.5 arc-seconds, and were then re-aggregated while preserving sub-pixel information, such as the relative extent of a polygon within the 15 arc-second pixel area of HydroSHEDS. This conversion method was applied, for example, to the high-precision data of lakes, reservoirs, and glaciers.

Due to different interpretations of the global coastline, the land extents of the input attribute grids typically exhibited slight mismatches in comparison to the spatial extent of the HydroSHEDS land mask, both over- and undershooting it (i.e., showing some pixel values in the ocean while lacking others on land). To prevent the creation of void attributes for coastal sub-basins, all resulting input grids were expanded or clipped to the HydroSHEDS land mask, which represents all global landmasses except Antarctica. If pixels in the original data appeared as ‘NoData’ on land areas of HydroSHEDS, these gaps were filled by allocating the value of the nearest existing pixel based on Euclidean distance. Some exceptions were made for particular attributes such as for elevation and population for which all pixels with missing values along the coast were substituted with zero instead of extending the value of the nearest neighbor. In contrast, if pixels in the original data were located within ocean areas of HydroSHEDS, they were removed from the final grid, i.e. set to ‘NoData’. A particular exception was made for population data as large numbers of people inhabit coastal areas and waterfront cities, thus the removal of pixels beyond the HydroSHEDS coastline would lead to a significant underestimation of population totals in the output grids. To avoid this loss, the population counts in pixels outside of the HydroSHEDS land mask were added to the nearest coastal pixel on land.

Besides mismatches along the coastlines, some original attribute datasets contained voids within the land mask of HydroSHEDS, or data were absent for small remote islands. Small gaps were automatically filled using ‘nearest neighbor’ interpolation. However, to avoid unsupervised allocation over long distances, all original ‘NoData’ areas that were more than 10 pixels (~5 km) away from existing attribute data were flagged and inspected manually to make decisions on a case by case basis. For example, the nationalities of some remote islands (more than 200 km away from the mainland) were manually assigned by looking up alternative sources. In some instances, large ‘NoData’ areas were retained in the attribute grids, e.g. if they covered all of Greenland or large deserts regions.

### Calculation of sub-basin and river reach statistics (‘local’ statistics)

After preprocessing the hydro-environmental source data, each resulting attribute grid was aggregated per sub-basin and per river reach and joined as an individual attribute column to the vector layers of HydroBASINS and HydroRIVERS. Different aggregation methods and statistics were applied as described below (additional specifications for individual attributes are provided in the Technical Documentation). Calculations were performed using the ‘Zonal Statistics’ tool of ESRI’s ArcGIS 10.4 software package^31^ embedded in customized batch scripts. The zonal statistics tool produces spatial summary statistics, including mean, majority, sum, maximum, and minimum, by performing calculations on cells from a value grid (i.e., the hydro-environmental attribute grid) within the unique spatial units of a zone grid. These zones are defined by cells with the same value (i.e., the unique sub-basin and river reach identifier codes). For the zonal statistics calculations, the sub-basin polygons and river reach line segments were applied in the native grid format of HydroSHEDS rather than in their converted vector representation to ensure proper alignment with the resolution and extent of the preprocessed attribute grids. After zonal statistics were derived, the resulting statistics were appended to the vectorized sub-basin polygons and river reach line segments via their unique identifier codes (IDs).

**Fig. 5:**
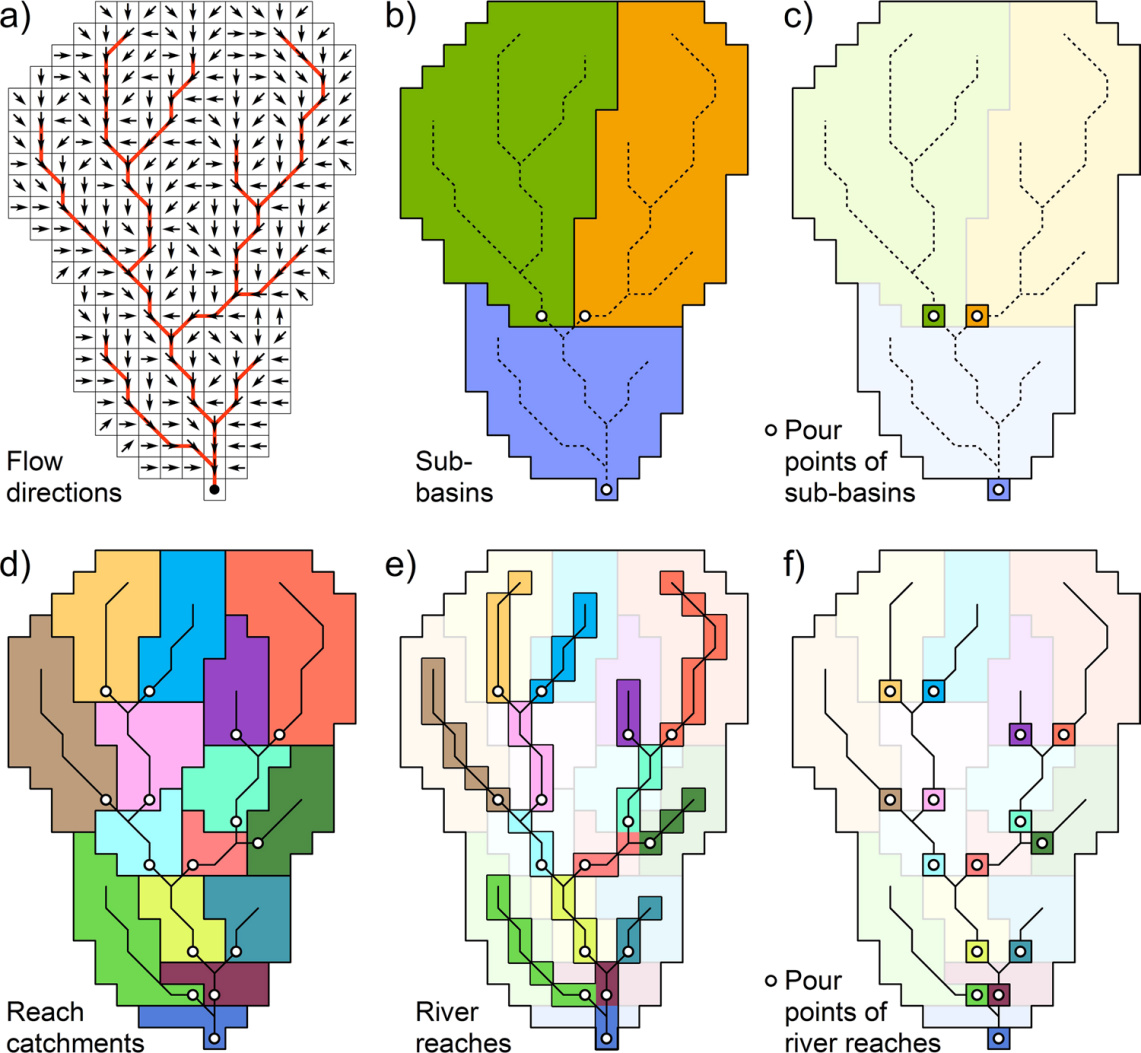
Different spatial aggregation units used for the extraction of sub-basin and river reach attributes of HydroATLAS. Panel (a) shows the flow directions of every pixel from which the river network (red lines) and sub-basins are derived. Other panels show the spatial zones of: (**b**) sub-basins; (**c**) sub-basin pour points; (**d**) reach catchments; (**e**) river reaches; and (**f**) reach pour points. Individual zones are identified by different solid colors, while light background shades are for orientation only.

Various zoning options were applied to derive specific attribute statistics in different (or multiple) ways, depending on the nature of the attribute variable. Figure 5 shows the relationship between the original flow directions and river network (Fig. 5a), as well as the derived spatial zones that were used to represent sub-basins and river reaches (Fig. 5b–f). For sub-basins, two alternative zones exist: (i) all cells that describe the entire sub-basin (Fig. 5b); or (ii) only the single cell that represents the pour point of the sub-basin, i.e. the most downstream pixel within the sub-basin before draining into the next sub-basin or the ocean (Fig. 5c). In contrast, three alternative zones exist for river reaches: i) all cells that form the contributing catchment of the river reach (termed ‘reach catchment’; note that reach catchments are different from sub-basins) (Fig. 5d); (ii) all cells that describe the river reach itself (Fig. 5e); or (iii) only the single cell that represents the pour point of the river reach (Fig. 5f).

The specific zones and statistics that were applied to extract each individual attribute are reported in the Technical Documentation of HydroATLAS. For example, some attributes are well suited to be calculated as the average or sum within the entire sub-basin or reach catchment (Fig. 5b,d), such as mean elevation or total population counts, respectively. Yet for other attributes using the entire sub-basin or reach catchment as the zone does not deliver a meaningful metric. For instance, a sub-basin typically contains pixels that span a wide range of possible discharge values, ranging from very small headwater streams originating at the edge of the sub-basin, to mainstem rivers traversing through the sub-basin with discharges that are orders of magnitude larger. Given this extreme heterogeneity, it is more meaningful to use a single, clearly defined pixel within the sub-basin as a representative location (i.e. zone) to extract discharge values. Hence, in BasinATLAS the representative discharge of a sub-basin is defined as the discharge that leaves the sub-basin at the pour point location (Fig. 5c), typically identical or close to the maximum discharge within the sub-basin; although exceptions occur, for example in arid regions where discharge can decrease along the river course. For the same reason of internal heterogeneity, the single-cell pour point approach is the most adequate option to represent any ‘upstream’ accumulation of an attribute (see next section below). Similar considerations as presented for the sub-basin example above apply for the river reaches of RiverATLAS, i.e. discharge values were extracted at the reach pour point (Fig. 5f) and other attributes were calculated using the most meaningful zoning method and statistic. The additional zone of the river reach itself (Fig. 5e) is useful for attributes such as the stream gradient.

It is important to note, however, that the suitability or meaningfulness of a variable and its zone may differ between sub-basin and river reach scales, and therefore the interpretation and use of the provided information remains a user’s choice. For instance, the percent forest cover within a sub-basin may represent a key hydrologic characteristic for the local conditions of that sub-basin, yet for a river reach, which is influenced by the larger drainage network upstream, the percent forest cover in the entire upstream watershed may be a better descriptor of its hydrologic condition.

### Calculation of ‘upstream’ statistics

Sub-basin and river reach statistics allow for a characterization of ‘local’ hydro-environmental conditions, such as the forested area within a sub-basin or within a reach catchment. Due to the hydrologic connectivity of the river network and associated sub-basins, however, many characteristics are better suited to an upstream perspective where the entire contributing watershed is taken into account. For example, if an application wanted to model the water temperature of a river reach, this would depend both on the conditions at the reach itself (e.g., ambient air temperature, local vegetation cover) and on conditions that originate in the contributing headwater areas that are connected to this river reach (e.g., air temperature or existing snow cover in the upstream mountain regions). The latter conditions can be described with upstream statistics, such as the average air temperature or the total glacier, snow, or forest extent in the entire upstream watershed that contributes to the river reach. In fact, it is the very nature of fluvial systems that they depend both on local conditions, defined by the immediate neighborhood that the river runs through, and by the conditions of the entire contributing upstream watershed which can include parts that are far away.

To allow for the duality of both local and upstream perspectives, HydroATLAS offers pre-calculated upstream statistics for many of its characteristics. Upstream perspectives are not provided for attributes where these calculations are not meaningful, such as for ‘minimum elevation’ (as the local minimum elevation of a sub-basin or river reach is identical to the lowest elevation of the entire upstream watershed), or for local attributes with an already inherent upstream perspective, such as river discharge. All upstream watershed statistics in HydroATLAS are extracted at the pour point location of sub-basins and river reaches.

The upstream perspective is particularly useful and fitting for river systems, as presented by the line segments of RiverATLAS. All parts within a river reach are affected by the larger upstream watershed as it drains towards and through it. For example, a river reach can be correctly described as having 20% snow cover in its upstream watershed. In contrast, the application of the upstream concept is more abstract for the sub-basin polygons of BasinATLAS. Each single sub-basin may represent a very heterogeneous mix of upstream influences: first, it will contain some pixels that represent the main river, which in analogy to a river reach is indeed affected by the sub-basin’s larger upstream watershed. But second, there are also pixels that represent very different locations within the sub-basin, including small tributaries or land next to the main river. These different locations are not affected by the larger upstream watershed of the sub-basin but only by their own individual contributing watersheds. So while the main river within the sub-basin may be affected by 20% snow cover upstream, all tributaries within the sub-basin may not be affected by snow cover at all. This spatial complexity mandates a careful interpretation of the suitability of upstream attributes before using them in intended applications.

Upstream values were calculated with the standard ‘Flow Accumulation’ tool of ESRI’s ArcGIS 10.4 software package^31^ to accumulate all upstream pixel values of an attribute grid along the drainage direction map of HydroSHEDS. In order to produce upstream averages, a correction was performed to account for the latitudinal distortion in pixel sizes due to the applied geographic projection: each pixel value was first multiplied by its individual pixel area and the accumulated sum of multiplied values was then divided by the accumulated sum of pixel areas to derive an area weighted average for the watershed. In a similar way, the upstream extent of an attribute (in percent coverage), such as percent forest cover, was calculated by dividing the total area of the attribute in the upstream watershed by the total watershed area, using latitude-corrected pixel areas.

Future versions of HydroATLAS are anticipated to include attributes with an upstream perspective where either distance weighting or runoff weighting will be applied. In distance weighting, every pixel is multiplied by a weight depending on their upstream distance, allowing for placing more emphasis on near versus far influences. In runoff weighting, every pixel is multiplied by a weight representing the local runoff amount, allowing for reducing or eliminating the influence of upstream areas that do not contribute much or any water to the downstream flows.

## Data Records

All hydro-environmental attributes available in version 1.0 of the HydroATLAS database, as well as their sources, are listed in Table 2. Most attributes with a time component (i.e. based on time series data) are provided as long-term annual averages in the attribute table of HydroATLAS, while some also include a monthly climatology, i.e. long-term monthly averages.

Each attribute offered in HydroATLAS is identified by a unique 10-character column name. More explanations and details on the syntax of the column names and other specifications pertaining to each attribute and its associated data source are provided in the Technical Documentation that is part of the HydroATLAS database (also available at http://www.hydrosheds.org/page/hydroatlas). In particular, the Technical Documentation includes a browsable catalog and overview maps for all available variables.

### Data format and distribution

All derived hydro-environmental attributes are provided in attribute tables associated with the sub-basin polygons of HydroBASINS and the river reach line segments of HydroRIVERS, respectively. Sub-basin characteristics were calculated for all Pfafstetter sub-basin levels, resulting in 12 individual multi-column attribute tables for the sub-basin polygons forming BasinATLAS. Only one multi-column attribute table was derived for the river reaches forming RiverATLAS.

HydroATLAS data are publicly available for download at http://www.hydrosheds.org/page/hydroatlas and as a static copy at the *figshare* data repository^32^. All map and data layers, including attribute tables, are offered in ESRI© Geodatabase and Shapefile formats. The data is projected using a Geographic Coordinate System based on the World Geodetic System 1984 (GCS_WGS_1984). The attribute table can also be accessed as a stand-alone file in dBASE format which is included in the Shapefile format. All data is distributed with an accompanying Technical Documentation.

## Technical Validation

The data compendium of HydroATLAS does not create new data from scratch but rather re-formats existing source data into the geospatial frameworks of HydroBASINS and HydroRIVERS. Unless specified otherwise, all source data are used “as is”, i.e. without modification except for disaggregation and aggregation processes, as well as the downstream accumulation along the drainage direction map of HydroSHEDS. Validation of the quality of original datasets remains with the source publications or documentations as cited in HydroATLAS.

The quality and limitations of the underpinning hydrographic framework of watersheds, river networks and drainage directions are discussed in the Technical Documentation of HydroSHEDS and related products (see http://www.hydrosheds.org). The choice of various specifications, such as the pixel resolution of 15 arc-seconds, the sub-basin breakdown by Pfafstetter levels, and the thresholds for the delineation of streams (see Methods) is in alignment with previous global applications of the HydroSHEDS product^19,26^ to ensure compatibility of HydroATLAS with existing studies, data, and results. The general aim of these choices is to provide data at very high spatial resolution, yet without exceeding the limits of accuracy and reliability of the underpinning global datasets, and without the need of exceptional (super)-computing facilities for users to process the data. Thresholds are also designed to deliver consistent geometric configurations. For example, in regard to the stream threshold settings, the chosen pixel size of 15 arc-seconds (~500 m) in combination with an upstream area threshold of 10 km^2^ produces streams once the catchment size exceeds about 40 pixels, ensuring that a dense river network is delineated even in arid and semi-arid regions. The complementary discharge threshold of 0.1 m^3^s^−1^ results in slightly increased river densities in humid regions as streams start to be drawn at even lower pixel limits. However, to exceed the applied discharge threshold within a single 15 arc-second pixel, an annual average runoff of approximately 12,000 mm would be needed. As even the most humid regions in the world reach only about half of this annual runoff^33^, the chosen pixel size and discharge threshold avoid that rivers start in areas smaller than one pixel, thus ensuring a river network that is geometrically sound and consistent in all regions globally.

As described in the Methods section, in order to limit distortions and avoid the introduction of bias, the disaggregation and aggregation steps applied for the generation of HydroATLAS refrain, as much as possible, from spatial interpolation methods. If original data needed to be re-projected, the ‘nearest neighbor’ approach was applied to avoid modification of original values. Global statistics and totals of the original data are thus preserved in HydroATLAS, with possible minor distortions along the global ocean coastline due to mismatching land-water masks in the different source datasets. In a specific data preprocessing step, human population counts were shifted onto the land mask of HydroSHEDS to prevent underreporting. This correction reduced the difference between total global population in HydroATLAS and the original source^34^ to only 0.07%; the remaining error being caused mostly by the omission of Hawaii on the HydroSHEDS land mask.

The use of ‘majority’ statistics, such as the assignment of the dominant land cover class to a sub-basin, can introduce statistical bias due to an issue known as ‘modifiable areal unit problem’ (MAUP)^35^, which can lead to different majority results when the same data is aggregated at different spatial scales. This problem needs careful consideration by the user before applying the results. For example, if a forest cover expands over 100% in one sub-basin and 10% in an equally sized neighboring sub-basin (with the remaining 90% being grassland), only one of the two sub-basins (half the total area) will show ‘forest’ as its majority land cover; but if the two sub-basins are lumped at the next coarser watershed scale, then the entire area will be dominated by forest (as 55% of the combined area is forest). This problem increases in complexity if multiple classes are present. As a general trend, the aggregated data will become less varied and more similar, i.e. frequent land cover classes will increasingly dominate at coarser scales at the cost of rare land cover classes which get subdued.

Given that country statistics are the intended output of many assessments, a particularly important example of the problematic and scale-dependent interpretation of ‘majority’ attributes is presented in the association of each sub-basin to a country. For countries with boundaries that are not crossed by sub-basins or rivers, such as Australia or any island nation, the country association of each sub-basin remains correct over multiple scales. In contrast, at land borders where rivers and sub-basins do extend over multiple countries, the majority association can change based on scale. For example, while the smaller headwater sub-basins of the Amazon Basin in the Andes are correctly associated to Ecuador, Bolivia, and Peru at finer resolutions, they are successively lumped into the larger sub-basins of the Amazon at coarser scales and are ultimately associated with Brazil due to its spatial majority at the largest basin scale. To quantify the increasing uncertainties caused by these majority associations across scales, Table 3 provides an overview of resulting errors for selected countries (a full list of all countries is available in Supplementary File 1). Results show that smaller countries tend to be affected by larger and more arbitrary omission and commission errors, and that errors grow for coarser scales (i.e. larger sub-basins). In comparison, the smaller reach catchments show only minor distortions in global average. Given these findings, users need to give careful consideration to inherent uncertainties before interpreting derived country statistics, particularly at coarser scales.

**Table 3:** Omission and commission errors for country associations in HydroATLAS. Omission errors represent parts of the country that were falsely assigned to another country; commission errors represent parts of other countries that were falsely assigned to the country. Values are in percent of the country’s own area. Sub-basin scales are based on Pfafstetter levels where larger numbers represent increasingly smaller sub-basin breakdowns. Levels 1–3 are not listed but show increasingly high and arbitrary errors. Selected countries are randomly chosen to represent different sizes and are sorted by area; a full list of all countries is available in Supplementary File 1.

Country	Area(10^3^ km^2^)	Error (%)	Sub-basin scale level									Reach catchment
			4	5	6	7	8	9	10	11	12	
Burundi	27.0	Omission	100.0	100.0	31.6	14.8	9.3	8.9	6.6	5.4	5.4	2.4
		Commission	0.0	0.0	50.9	48.2	17.7	7.2	5.5	6.1	6.1	2.7
Switzerland	41.7	Omission	100.0	36.3	36.3	24.0	7.1	3.6	3.3	2.4	2.4	1.5
		Commission	0.0	16.7	16.7	3.9	7.1	6.3	4.2	4.2	4.2	1.5
Austria	83.8	Omission	3.9	11.2	13.3	11.6	9.4	3.8	2.2	2.3	2.3	1.0
		Commission	204.9	45.1	28.3	12.3	4.7	3.3	2.5	2.3	2.3	1.1
Nepal	147.7	Omission	100.0	27.3	25.5	11.6	5.3	2.8	2.0	1.9	1.9	0.9
		Commission	0.0	76.9	19.1	14.1	6.4	4.3	2.4	2.2	2.2	0.8
Laos	229.9	Omission	31.9	20.5	14.6	7.2	4.8	2.4	1.6	1.5	1.5	0.6
		Commission	22.8	29.6	12.5	7.3	2.9	2.6	1.9	1.7	1.7	0.8
France	550.8	Omission	28.1	3.3	4.7	1.3	0.9	0.5	0.5	0.5	0.5	0.2
		Commission	8.2	15.0	4.4	2.7	0.7	0.6	0.3	0.3	0.3	0.2
Bolivia	1,083.4	Omission	20.7	15.8	8.1	6.1	3.8	1.5	1.0	0.8	0.8	0.4
		Commission	76.7	30.1	19.5	4.6	1.7	1.4	1.0	0.9	0.9	0.4
DR Congo	2,328.2	Omission	15.4	8.7	6.5	3.1	1.6	0.7	0.6	0.6	0.6	0.3
		Commission	20.5	12.0	4.0	2.7	1.2	0.9	0.6	0.5	0.5	0.2
India	3,156.0	Omission	6.2	8.2	4.3	2.1	1.2	0.8	0.5	0.5	0.5	0.2
		Commission	16.5	6.4	4.4	2.2	1.5	0.8	0.5	0.5	0.5	0.2
Australia	7,700.8	Omission	0.0	0.0	0.0	0.0	0.0	0.0	0.0	0.0	0.0	0.0
		Commission	0.0	0.0	0.0	0.0	0.0	0.0	0.0	0.0	0.0	0.0
Canada	9,996.5	Omission	3.3	1.8	1.5	0.8	0.4	0.2	0.1	0.1	0.1	0.1
		Commission	3.9	2.6	0.8	0.6	0.4	0.2	0.1	0.1	0.1	0.1
Russia	16,946.3	Omission	3.2	1.8	1.4	0.7	0.3	0.2	0.2	0.2	0.2	0.1
		Commission	4.4	2.3	1.0	0.7	0.4	0.2	0.2	0.2	0.2	0.1
World*	**134,716.0**	**Omission**	**42.9**	**26.9**	**13.4**	**7.4**	**4.1**	**2.4**	**1.7**	**1.6**	**1.6**	**0.7**
		**Commission**	**19.3**	**16.2**	**13.3**	**6.8**	**3.7**	**2.4**	**1.7**	**1.6**	**1.6**	**0.7**

*All 169 global countries that exceed 10,000 km^2^ in their individual area (very small countries < 10,000 km^2^ show increasingly arbitrary errors).

Another potential error can occur in coastal sub-basins that represent multiple lumped (small) coastal rivers and their individual watersheds draining into the ocean. As these coastal sub-basins have multiple pour points along the shoreline rather than a single one, pour point statistics such as ‘average’ may deliver incorrect results as each pour point’s value is weighted equally rather than by contributing watershed area. In contrast, other statistics such as ‘sum’ or ‘maximum’ will be correct.

For many hydrological applications, the runoff and discharge estimates provided as part of the HydroATLAS database will be particularly important. Given the inherent uncertainties of global hydrological models, ideally an ensemble of different model runs should be provided. However, to our knowledge no global hydrological model results are publicly available below 5 arc-minute spatial resolution, and any downscaling of discharge information from coarse to fine resolution presents a major technical challenge. Hence only one set of runoff and discharge estimates is offered in version 1.0 of HydroATLAS. Like all other attribute data, this information was provided by an existing source and was only reformatted to fit with HydroATLAS. Yet given its importance we conducted a baseline evaluation of the discharge data. The estimates of long-term (1971–2000) discharge averages provided in HydroATLAS were derived through a geospatial downscaling procedure^26^ from the 0.5 degree resolution runoff and discharge layers of the global WaterGAP model^33^ (version 2.2 as of 2014), a well-documented and validated integrated water balance model. After downscaling, the global total river flow into all oceans matched the original flow as modeled in WaterGAP within an error margin of 0.13%, indicating no significant distortion of large-scale totals due to the downscaling process. In addition, a validation of the downscaled discharge estimates against observations at 3,003 global gauging stations^36^, representing river sizes from 0.004 to 180,000 m^3^s^−1^, confirmed good overall correlations for long-term average discharges (R^2^ = 0.99 with 0.2% positive bias and a symmetric mean absolute percentage error sMAPE of 35%, improving to 13% for rivers ≥100 m^3^s^−1^).

## Usage Notes

HydroATLAS offers a large variety of hydro-environmental attributes intended for a broad range of user applications. It remains the user’s responsibility to decide whether certain attributes, statistics, or scales are meaningful and appropriate. For example, the association of a large river basin to a single country based on spatial majority may be adequate for a basin that is entirely or mostly within the country, but can be highly misleading for a transboundary basin spanning many countries. Similarly, the association of coarser scale attributes, such as national GDP values, to small sub-basins or river reach catchments may be meaningful for statistical assessment purposes, yet will not realistically represent small-scale spatial patterns. Careful interpretation is also mandated if users choose to apply the ‘upstream’ attributes offered in BasinATLAS, as these attributes are representative only for the main river draining the sub-basin rather than the entire sub-basin area (see related explanations in the Methods section).

Beyond the existing attribute columns contained in HydroATLAS, users can extract a variety of inherent information by applying their own post-processing algorithms and cross-calculations. For example, attributes can be analyzed by comparing results across different scales, such as identifying the number or area of small sub-basins (e.g., Pfafstetter level 10) that exceed a given thresholds, such as a temperature limit, within a larger sub-basin (e.g., Pfafstetter level 6). Similarly, attributes can also be summarized by other attributes, such as average runoff per country, or runoff per land cover type; these types of cross-correlations are best performed at finer sub-basin scales to increase spatial congruence. Finally, attributes can also be normalized using the existing information of multiple columns. For example, discharge can be divided by upstream watershed area in order to calculate ‘specific discharge (per km^2^)’; or by upstream population numbers in order to calculate ‘water availability per person’.

As both BasinATLAS and RiverATLAS are derived from the same underpinning hydrography of HydroSHEDS, they are mutually linkable via their uniquely defined spatial relationship whereby every river reach falls within a sub-basin (many-to-one relationship). Similarly, HydroATLAS is fully compatible with other raster and vector datasets that are built from, or linked to, the hydrographic framework of HydroSHEDS, such as the lake and reservoir polygons of the HydroLAKES database^37^ as well as a growing range of aquatic species compilations including continental maps produced by IUCN^25,38^.

Intensive efforts have been made to verify the licenses of the underpinning source datasets, and specific permissions were obtained from individual authors if needed, in order to release all derived attribute columns of HydroATLAS (version 1.0) under either a Creative Commons Attribution 4.0 International License (CC-BY 4.0) or an Open Data Commons Open Database License (ODbL 1.0), both permitting reuse of the data for any purpose including commercial. HydroATLAS users are requested to honor the individual reference suggestions of the source data providers; hence citations and acknowledgements should be made to both the original data source(s) and the HydroATLAS compendium. For example, the following template illustrates a reference to precipitation data sourced from HydroATLAS: “Precipitation data from the WorldClim v1.4 database (Hijmans *et al*. 2005) has been used in the spatial format of HydroATLAS v1.0 (Linke *et al*. 2019).” Detailed information regarding the license and reference(s) for each attribute column is provided in the Technical Documentation of HydroATLAS and in Table 2.

## Supplementary information

Supplementary File 1

## Data Availability

All data processing steps were performed using native tools and/or customized batch processing within ESRI’s ArcGIS 10.4 software package^31^ in a dedicated computing setup (64-bit processing). The two core tools applied were ‘Zonal Statistics’ and ‘Flow Accumulation’. To support repetitive tasks of this work, a multitude of adjusted batch routines were developed as needed, mostly defining input and output path names for the standard tools and to handle internal object IDs. No stand-alone programming code was created that allows automatic processing of new data into the format of HydroATLAS. This is in alignment with the premise of our work, i.e. to produce standardized data by applying tedious, individual, and customized GIS steps specific to every input dataset so that other user do not have to repeat these time-consuming manual iterations.
